# Direct brain recordings reveal implicit encoding of structure in random auditory streams

**DOI:** 10.1038/s41598-025-98865-5

**Published:** 2025-04-27

**Authors:** Julian Fuhrer, Kyrre Glette, Jugoslav Ivanovic, Pål Gunnar Larsson, Tristan Bekinschtein, Silvia Kochen, Robert T. Knight, Jim Tørresen, Anne-Kristin Solbakk, Tor Endestad, Alejandro Blenkmann

**Affiliations:** 1 RITMO Centre for Interdisciplinary Studies in Rhythm, Time and Motion, University of Oslo, Oslo, Norway; 2 Department of Informatics, University of Oslo, Oslo, Norway; 3 Centre for Precision Psychiatry, Division of Mental Health and Addiction, University of Oslo and Oslo University Hospital, Oslo, Norway; 4 Department of Neurosurgery, Oslo University Hospital, Rikshospitalet, Oslo, Norway; 5 Cambridge Consciousness and Cognition Lab, Department of Psychology, University of Cambridge, Cambridge, UK; 6 ENyS-CONICET-Univ Jauretche, Buenos Aires, Argentina; 7 Helen Wills Neuroscience Institute and Department of Psychology, University of California, Berkeley, USA; 8 Department of Psychology, University of Oslo, Oslo, Norway; 9 Department of Neuropsychology, Helgeland Hospital, Mosjøen, Norway

**Keywords:** Statistical learning, Pattern detection, Predictive coding, High-frequency activity, MMN, Auditory system, Cognitive neuroscience, Learning and memory

## Abstract

The brain excels at processing sensory input, even in rich or chaotic environments. Mounting evidence attributes this to sophisticated internal models of the environment that draw on statistical structures in the unfolding sensory input. Understanding how and where such modeling proceeds is a core question in statistical learning and predictive processing. In this context, we address the role of transitional probabilities as an implicit structure supporting the encoding of the temporal structure of a random auditory stream. Leveraging information-theoretical principles and the high spatiotemporal resolution of intracranial electroencephalography, we analyzed the trial-by-trial high-frequency activity representation of transitional probabilities. This unique approach enabled us to demonstrate how the brain automatically and continuously encodes structure in random stimuli and revealed the involvement of a network outside of the auditory system, including hippocampal, frontal, and temporal regions. Our work provides a comprehensive picture of the neural correlates of automatic encoding of implicit structure that can be the crucial substrate for the swift detection of patterns and unexpected events in the environment.

**Supplementary Information:**

The online version contains supplementary material available at 10.1038/s41598-025-98865-5.

## Introduction

Efficient encoding of patterns in ongoing sensory input is critical for survival in an ever-changing environment. Pattern encoding involves continuously updating internal representations of the environment based on statistical structures derived from the sensory signal^1–7^. The brain is not inherently aware of the underlying structures in the environment, and potential regularities in the sensory stream must be assessed continuously according to previously encoded regularity^8–10^. Sensitivity to conditional regularity between events has been observed in humans^11–21^ and animals^22,23^. Because events in the environment rarely occur independently, this pattern extraction is necessary for the fast and efficient processing of sensory information and optimal behavior.

A mathematical representation of such conditional regularity is transitional probabilities (TPs). TPs describe how likely one event predicts another. That is the ratio of the directional co-occurrence of events given their frequency^3,24–26^. As an example, experimental studies in infants and adults have shown that the TPs between syllables constitute patterns that facilitate the identification of word-like units^11,26–30^, thus making TP encoding essential for language development^3,4,25,28,31–34^.

While the brain’s sensitivity to conditional regularities has been observed across sensory domains, the underlying mechanisms remain poorly understood^3,27,28,35–42^. Research indicates that statistical regularities’ perception and learning involve multiple brains, suggesting a distributed network rather than a single neural region performing this function^28,32,33,40,43,44^. Sensory modality-general areas, such as the prefrontal cortex and the hippocampus, alongside lower perceptual or modality-specific regions, are proposed to subserve this capacity. However, our knowledge of which brain regions contribute to these dynamic and adaptive processes is still limited^3,14,29,33,40,41,45,46^. These regions’ involvement depends on several factors, including the structure and complexity of the input (e.g., pure tones versus linguistic material or sequence duration) and whether learning is implicit or explicit^28,31,32,47^. Traditional neuroimaging techniques often provide a broad and less specific view of brain activity, which limits our ability to capture the nuanced and rapid neural processes involved in statistical learning^48^. This limitation hampers the sensitivity to more subtle TPs, underscoring the need for further exploration to disentangle the specific contributions and interactions of brain regions, especially under conditions of automatic learning without directed attention^33^. Also, understanding these underlying mechanisms could offer significant insights into how complex cognitive processes are supported by distributed neural networks.

Traditionally used as contrast sequences and often assumed to lack structure^42,49^, random sequences have the potential to reveal important neural dynamics in implicit statistical learning. Research indicates that even seemingly random sequences can contain latent structures that impact neural learning, showcasing the brain’s ability to adaptively interpret complex inputs^50,51^. Moreover, evidence suggests that humans can track the statistical structure of random auditory environments^52^. Similarly, cognitive biases like the gambler’s fallacy highlight how humans process randomness, expecting alternating patterns rather than repetitions. While these phenomena are well documented at the behavioral level, exploring their cortical underpinnings is necessary to understand better how the brain encodes and predicts random events^10,53–55^.

To address this knowledge gap, we hypothesized that a core function of the brain is to automatically and continuously encode TPs in an online fashion, implemented in a distributed manner through processes not requiring voluntary attention. Specifically, we investigated how different brain regions contribute to implicit statistical learning by exploiting intracranial electroencephalography’s (iEEG) high temporal and spatial resolution. We estimated the trial-by-trial information content of high-frequency activity (HFA; 75 to 145 Hz), a correlate of population neuronal spiking, from participants who were passively exposed to an auditory sequence of randomly occurring tones composed of standard or deviating tone types. First, we validated our information-theoretical approach on previous deviance detection literature. In the main analysis, we then analyzed this information content estimate against the dynamic TPs of the deviating tone types stemming from an ideal observer model. Our results reveal that the brain continuously encodes the TPs in a stream of random stimuli through a network that spans areas outside the auditory system, including hippocampal, frontal, and temporal regions. Remarkably, this automatic process occurs even without evident relations within the deviant stimuli or behavioral relevance. Our study offers unique insights into neural adaptability by elucidating how the brain processes statistical information beyond fixed probability structures. It specifically highlights the brain’s capability to process complex inputs without directed attention, with a focus on implicit learning and the dynamic nature of random sequences. This allowed us to identify the involvement of multiple brain regions in managing diverse and unpredictable auditory inputs, enhancing our understanding of the areas and neural mechanisms underlying statistical learning.

## Results

### iEEG unattended listening task

**Fig. 1:**
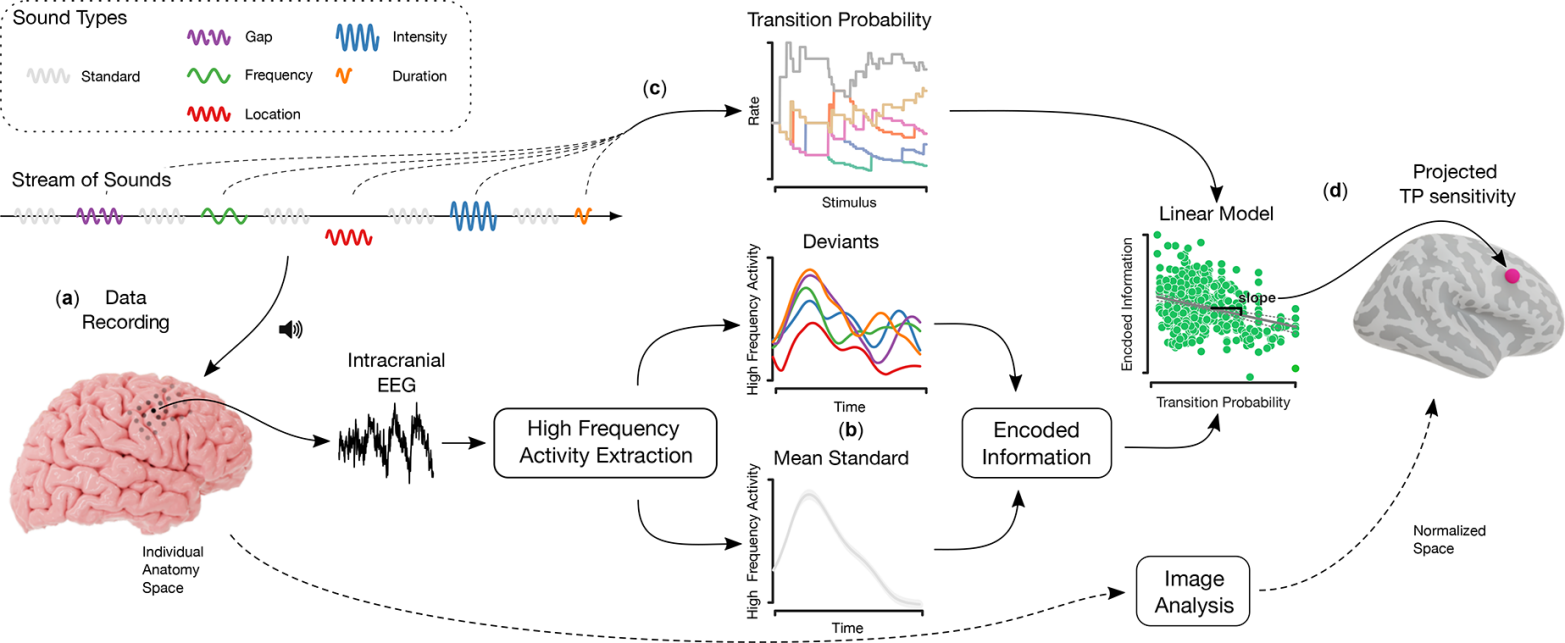
Overview of the analysis. (**a**) An unattended listening task was presented to participants while recording their event-related electrical brain activity through intracranial electrodes. High-frequency activity (HFA) responses to standard and deviant tones were extracted from the iEEG signals. (**b**) HFA responses to standard tones were then averaged to compute a channel-specific mean standard response. Differences in normalized encoded information between single-trial deviant and mean standard responses were computed using a compression algorithm. The higher the value of encoded information, the lower the similarity between the mean standard and a respective deviant tone response. (**c**) TP estimates were computed exclusively between deviants (disregarding the unimportant and static transitions involving standard tones). TP estimates were obtained from an ideal observer model, analyzing the deviating types within the stream of sounds. (**d**) In the next step, the main analysis was carried out by computing the linear correlation between encoded information and TP estimates of single trials. Finally, channel-specific slopes from the regression analysis were studied within regions of interest (ROIs) to incorporate data across subjects. Lastly, results were projected onto the normalized anatomical space for visualization (Fig. 3).

We collected intracranial EEG data from 22 patients with drug-resistant epilepsy who were implanted for clinical reasons (“Methods” and Table S1). Participants listened to a stream of sounds where standard tones were interleaved with deviant tones (inter-stimulus interval 500  ms). This multi-dimensional auditory oddball paradigm (Optimum-1^2^) contains deviant sounds that differ from the standard sounds in terms of either frequency, intensity, perceived sound-source location, a shortened duration, or a gap in the middle of the tone (probability P = 0.1 for each deviant type; Fig. 1, “Methods”). The stimulus set consisted of five standard tones and five deviant tones, with each of the five deviant types presented once in a random order within a block of ten sounds. In successive sets, the same deviant type did not repeat from the end of one to the beginning of another. For deviations in location, intensity, and frequency, two stimuli versions occurred (P = 0.5 for each deviant type): location left/right, intensity low/high, and frequency low/high. Together with the other two deviants, this resulted in eight potential deviants. This design keeps the frequency of occurrence of deviants stable throughout the experiment, though the likelihood of each deviant type being followed by another deviant type in the next iteration is constantly changing. Except for deviants varying in duration, all tones had a duration of 75 ms and were presented every 500 ms in blocks of 5 m consisting of 300 standards and 300 deviants. At the beginning of each block, 15 standards were played. During the recording, participants were asked not to pay attention to the sounds while reading a book or magazine. All participants reported that they focused on the reading material and did not attend to the tones or notice any patterns in the stimuli and completed 3 to 10 blocks, providing at least 1800 trials for analysis.

The recordings were manually cleaned by excluding noisy or epileptic channels or segments from the analysis, resulting in a total of 1078 channels (mean: 48, range: 12 to 104). HFA was then reliably extracted from a total of 785 channels within cortical or subcortical structures, and HFA event responses (trials) were evaluated in the 0 to 400 ms time window following the sound onset.

### Encoded information peaks in primary and secondary auditory cortices

We used encoded information to obtain a unique perspective on the neurophysiological recordings, leveraging information theory. The encoded information computed between two signals indicates their similarity in terms of information content^56–61^. Information or informational content, in this context, refers to the complexity or the amount of data contained within a signal, which can reflect how ordered or random a signal is. We computed the encoded information of deviant tone responses relative to standard tone responses. Smaller values of *encoded information* suggest that the information content in deviants is similar to that in standards. Greater values, on the other hand, indicate a larger amount of encoded information in the responses to deviants compared to standards. Using encoded information adds benefits compared to more standard measures based on power or amplitude changes. It is not bound to a single type of data, is model-independent (i.e., does not require assumptions about the data itself), and can capture nonlinear interactions^56,57,60–62^. As primary and secondary auditory areas are known to be more sensitive to deviant sounds than non-auditory areas, we hypothesized this should be reflected in higher encoded information^1,35,63–71^

**Fig. 2:**
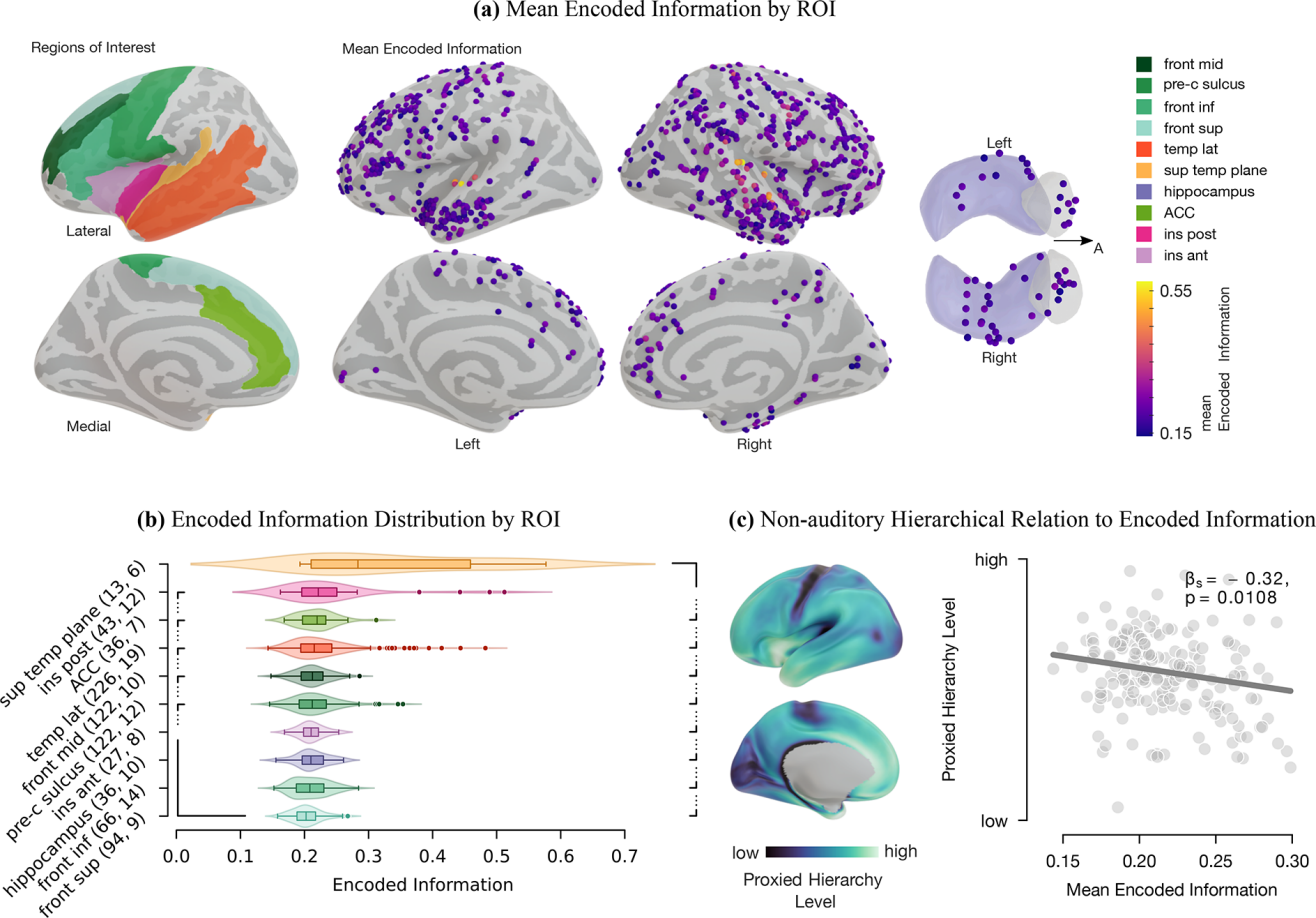
Mean encoded information-based deviance detection analysis results. (**a**): Left: ROIs on the inflated brain model (Table S2 for full region labels). Middle: Lateral and medial view of the mean encoded information (color-coded) across 22 subjects. Each channel, represented by a sphere, was projected onto the inflated brain surface model. Right: transverse plane of the amygdala (gray) and hippocampus (purple). “A” stands for the anterior direction. (**b**) Distribution of the ROIs’ encoded information. The number of channels (first) and subjects (second) for each ROI are in the axis labels. The nested brackets left or right of the violin plots indicate a significant difference between median values. (**c**) In non-auditory areas, proxied hierarchy levels correlated with mean encoded information (each dot represents a channel). The lower the cortical hierarchy, the greater the mean encoded information.

More precisely, to compute encoded information, we estimated the single-trial information content of each deviant tone HFA response in relation to the information content of the mean HFA responses to standard tones. Accordingly, the information contained in standard responses was used as a reference value to measure the information content in deviant responses, which yielded a normalized measure of *encoded information* for each single-trial deviant tone response (Fig. 1, bottom; “Methods”). To systematically evaluate the involvement level across the cortex, we defined regions of interest (ROIs) that typically engage in auditory processing and statistical learning tasks^1,28,32,33,42^, comprising temporal, frontal, insular, peri-central sulci, and ACC cortices, as well as the hippocampus (Fig. 2a, Table S2). We then compared the median *encoded information* across the ROIs (Fig. S1). This comparison aligned our information-theoretical approach by evaluating how our results coincide with existing deviance detection literature. The greatest median *encoded information* values were observed in primary and secondary auditory cortices (superior temporal plane, insula posterior, and temporal lateral ROIs), suggesting that core aspects of deviant processing locate there (Fig. 2b , two-tailed pairwise Mann–Whitney–Wilcoxon tests, FDR corrected, $\text{p}\le$ 1.12e−2, $|\text{z}|\ge 2.5$). Each ROI’s median encoded information was significantly greater than zero (one-tailed Wilcoxon signed-rank test, FDR corrected, $\text{p}\le$ 1.22e−4, $\text{z}\ge 4.53$). Altogether, these results indicate that the encoded information in the responses to deviants reflects the local sensitivity of specific brain areas to unexpected events in accordance with previous studies on deviance detection^63,69,70,72^. Consequently, these results support our use of encoded information as a valid method. In^56,57^, this method was further tested and validated by comparing it to traditional statistical techniques and using simulated data, showing that it effectively detects complex changes in brain signals. By examining various datasets, we confirmed that encoded information is a reliable and effective tool for studying how the brain processes information. Additionally, we examined the sensitivity to specific deviant types across ROIs. Statistical analysis only identified significant differences in the encoded information of specific deviant types in the superior frontal area. The statistically significant differences were between the deviant types of “location left”, “intensity up”, and “frequency down” to “gap” (Fig. S5, two-tailed pairwise Mann–Whitney–Wilcoxon tests, FDR corrected, $\text{p}\le {5.30e{-}4}$, $\text{z}\ge 3.5$).

### Encoded information is hierarchically organized

Previous animal and human studies indicate a hierarchical organization of brain regions underlying the detection of unexpected events^6,42,43,73^. In the light of deviance detection, we utilized a proxy measure of anatomical hierarchy to investigate to what extent this is reflected in the *encoded information* values across brain regions. Anatomical hierarchy can be defined as a global ordering of cortical areas corresponding to characteristic laminar patterns of interareal feedforward and feedback projections^5,74,75^. Proxied cortical hierarchy levels that quantify these projections across the cortex were obtained from open-access structural magnetic resonance imaging (MRI) datasets from the S1200 subject release (^76^; “Methods”). Methodological constraints in^76^ precluded the mapping of the hippocampus in the present analysis. Areas lower in the hierarchy (with predominantly feedforward projections) are primarily associated with primary sensory functions, whereas areas higher in the hierarchy are associated with higher cognitive functions^5,74,75^. For each contact point, hierarchy-level channel estimates were determined by taking the average value of all proximal points located in the vicinity of the contact point. We observed a significant negative correlation between the *encoded information* and the proxied cortical hierarchy levels (Fig. 2c ; linear mixed-effects model with random effects for subjects: $y=\beta _0+\beta _1 x+b_0+\epsilon$, with the log-transformed proxied hierarchy level *y*, the encoded information *x*, the random effect for subjects $b_0 \sim N(0,\sigma ^{2}_b)$ and the observation error $\epsilon \sim N(0,\sigma ^{2})$; $\beta _0=0.13$, 95% CI [0.07 0.18], $\beta _1=-0.32$, 95% CI [− 0.07, − 0.56], $\text{p}_{\beta _1}$ = 1.08e−2, $\sigma _b$ = 4.58e−2, 95% CI [4.10e−2, 5.11e−2], $\epsilon$ = 2.84e−2, 95% CI [1.81e−2, 4.45e−2]). Outliers values were defined as values being greater than three times the median absolute deviation (MAD) on both encoded information (0.3) and proxied hierarchy levels (0.04). Further, we focused on non-auditory areas and excluded respective auditory channels (superior temporal plane, insula posterior, and lateral temporal ROIs). This prevented the regression model from being solely driven by auditory regions with high encoded information values and low proxied hierarchical levels.

### Ensemble activity exhibits sensitivity to transitional probabilities

**Fig. 3:**
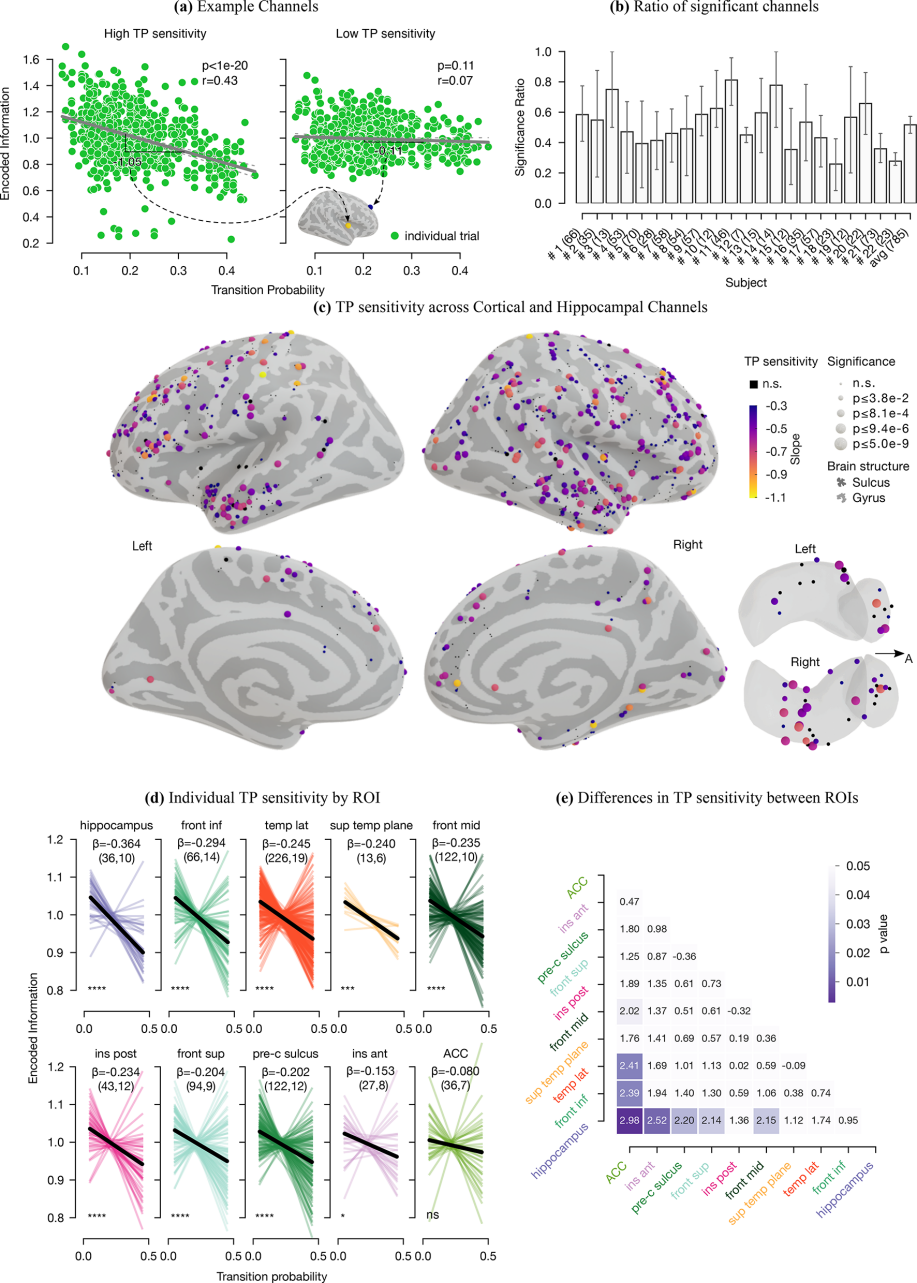
Implicit statistical learning of deviants’ TP courses. (**a**) Two example channels (high and low) TPs sensitivity between deviants, where each green dot is a deviant trial. TP sensitivity was estimated for each channel by robust linear regression between TPs and encoded information, normalized by channel mean. The slope shows regional sensitivity under a contact point to TP variation. Channel one has a negative slope of − 1.05, indicating that more frequent transitions lead to decreased encoded information. (**b**) Ratio of significant channels (number in brackets) across subjects. Error bars indicate the 95% CI across ROIs. (**c**) Spatial distribution of TP sensitivity across cortical and subcortical areas. Each sphere is a channel projected onto the inflated brain model surface with lateral and medial views of both hemispheres, plus superior views of the amygdala and hippocampus. Colors indicate TP sensitivity. Sensitivities greater than − 0.3 or within the first 25% of all values appear with the gradient’s lowest color. Sphere size represents p-values, divided such that each interval contains $\nicefrac {1}{4}$ of the p-value set. (**d**) TP sensitivity by ROIs, with channel-individual sensitivities shown. The median TP sensitivity is in black (see $\beta$ for value). Channel and subject numbers are in parentheses in the subtitles. All ROIs except ACC show significant median TP sensitivity (significance: “ns” p ${>0.05}$, *p ${\le 5e{-}2}$, p ${\le 1e{-}3}$, and ****p ${\le 1e{-}4}$). (**e**) Z-value matrix representing individual statistical differences of TP sensitivity between ROIs.

After validating our information theoretical measure on deviance detection literature, we investigated the brain’s sensitivity to TPs between deviant tones, examining whether areas beyond the auditory cortex contribute to the dynamic tracking of TPs. We incrementally (i.e., trial-by-trial) estimated TPs between adjacent deviants in the fashion of an ideal observer model (“Methods”). Given task-design constraints, TPs involving standard tones are static (“S$\rightarrow$D”/“D$\rightarrow$S”) or equal to 1 and disregarded. For instance, in a sequence “...S D$_3$ S D$_2$ S D$_4$ S D$_1$ S D$_2$...”, consisting of standard tones “S” and deviant tone types “D$_{1-4}$”, the transitions of interest are “D$_3$ $\rightarrow$D$_2$”, “D$_2$ $\rightarrow$D$_4$”, “D$_4$ $\rightarrow$D$_1$”, and “D$_1$ $\rightarrow$D$_2$”. At each given deviant event (trial), TP values were updated based on all previously presented deviant stimuli. Consequently, TPs dynamically evolved during the experiment. Note that finite streams, as opposed to infinite horizon streams, naturally entail temporally fluctuating patterns because of the alternating occurrence of deviants (Fig. 1, TP graph, “Methods”). We estimated the relationship between encoded information and the TPs of deviant tones through robust linear models. We thereby determined which brain area exhibits sensitivity to these temporal relations. Before regression, the trial-specific encoded information values were normalized by channel means to correct the encoded information that solely reflects auditory sound processing mechanisms (Fig. S6). This normalization approach aligns with the supplementary analysis on the relationship between channel-wise variance in Standard HFA and mean EI (Fig. S7), supporting the notion that variability in neuronal responses correlates with greater information encoding, thus highlighting the relevance of accounting for such variance. For each channel, the resulting regression slope was defined as the channel-specific *TP sensitivity*. *TP sensitivity* values indicated how sensitive the brain tissue around the channel was towards TPs between deviants in the stream of tones. Zero value *TP sensitivity* of a channel specifies that the encoded information in the deviant responses is not affected by the TPs of the events (i.e., null hypothesis), whereas lower values imply a higher impact. Figure 3a shows two example electrodes of high and low *TP sensitivity* (each green dot represents a deviant trial). In the analysis, 61.53% of the 785 channels across all subjects showed a significant *TP sensitivity* (Fig. 3b and Fig. S3, permutation-based test, FDR corrected). These channels tended to increase the amount of *encoded information* in the HFA response when the likelihood of an event occurrence decreased (low TP) and conversely decreased the *encoded information* for more expectable events (high TP). Notably, the *TP sensitivity* distributes over the brain (Fig. 3c). Therefore, we evaluated this distribution in terms of ROIs. Each ROI’s *TP sensitivity* except for the ACC were significantly lower than zero (one-tailed Wilcoxon signed-rank test, FDR corrected, $\text{p}\le$ 2.97e−2, $|\text{z}|\ge 1.89$), indicating that most ROIs were involved in the encoding of TPs (Fig. 3d). Importantly, our results were consistent across participants. Out of the 22 subjects, an average of 52.10% (95% CI [47.19 %, 57.04%]) showed a significant *TP sensitivity* across the ROIs (Fig. 3b and Fig. S4). Further analysis employing mixed-effects models detailed in Fig. S8 confirmed these results, showing robust TP sensitivity in these regions. Moreover, we studied differences in the *TP sensitivity* across ROIs, where the hippocampus and inferior frontal gyrus showed the highest sensitivity to TPs (Fig. 3e, two-tailed Mann–Whitney–Wilcoxon tests, $\text{p}\le$ 4.37e−2, $|\text{z}|\ge 2.02$).

## Discussion

We studied how humans passively listening to a multi-feature sequence of random sounds implicitly encode conditional relations between them. Crucially, we show that the auditory system embedded in a distributed hierarchical network continuously monitors the environment for potential saliency, maintaining and updating a neural representation of temporal relationships between events. This suggests that the brain constantly attempts to predict and provide structure from environmental events, even when unattended, not behaviorally relevant, and without any evident relation between them.

Participants demonstrated remarkable sensitivity to the TPs between deviant tones. Following Frost et al.’s^47^ definition of statistical learning (“all phenomena related to perceiving and learning any forms of patterning in the environment that are either spatial or temporal in nature”^47^), our findings suggest an implicit learning process in which TPs are internally inferred. On average, more frequent deviant transitions exhibited less encoded information in the HFA responses. Conversely, rarer transitions showed an increase in encoded information (Fig. 3a, c). Consequently, these results indicate an encoding of TPs, consistent with previous studies using more structured, stationary, or attentive stimuli in humans^4,11–21,25,42,46^ and non-humans^22,23,43^. Unique to our study is that we demonstrate the brain’s sensitivity to dynamic TP courses without directed attention in a randomly structured sequence of varied auditory deviant stimuli. The brain’s sensitivity to TPs within our random sequence suggests a more general and automatic mechanism that continuously encodes TPs between environmental events. This critical mechanism forms the basis of a statistical learning system wherein the brain integrates every event into an internal representation of the environment based on the statistical relationship between events. Since a priori the presence of patterns within stimuli is unknown, the brain might automatically encode their TP to detect potential structure and violations of such. Artificial grammar learning studies, where subjects learn patterns of nonsense words, confirm the relevance of this TP encoding in language learning^16,28,29,33,34,77^.

Following the notion of predictive coding, the encoded information in each deviant tone response can be interpreted as a bottom-up prediction error signal, i.e., the amount of information in each novel event not explained away by top-down prediction signals^5,7,43,78^. Consequently, low TP events, i.e., less expected events, elicited higher encoded information and, hence, larger prediction errors derived from less accurate predictions. Accordingly, this information is used in higher cortical areas to update internal models for future predictions. On the other hand, high TP events, i.e., more expected events, elicited a lower amount of encoded information. This generates smaller prediction error signals and smaller updates of the internal models. Internal representations of TPs between events are fundamental to building useful predictions of upcoming events rather than simpler frequentist representations^12,24^. However, studies investigating predictive processing rarely consider TPs. In statistical learning studies, on the other hand, a possible sensitivity to TPs is often examined, yet less extensively on a neurophysiological level. Based on iEEG, our study takes a step forward in both directions. By providing correlates of a vast and distributed network supporting implicit statistical learning, our results support the view that TPs might constitute a central statistic used by internal perceptual models at the core of implicit predictive processing and statistical learning.

Our results provide novel evidence that the encoding of acoustic transitions is anatomically distributed and not exclusively concentrated in auditory cortices (Fig. 3c). The automatic process of identifying temporal relationships is subserved by a network encompassing the hippocampus in concert with the inferior frontal, temporal, and insular cortices. Accordingly, by entailing multiple active brain regions, this network bundles together findings from various prior statistical learning^28,32^ and predictive processing^6,42^ studies.

Specifically, the hippocampus contributes most to the implicit transition encoding between salient events. In contrast to other areas, hippocampal responses indicate high sensitivity to TPs while having lower sensitivity to deviant tones (Figs. 2b and 3d). Accordingly, hippocampal activity may reflect a more generic context sensitivity to the events’ probabilistic structures, i.e., learning about event frequencies within a given structure instead of encoding actual deviating events^79^. Our results provide new evidence for the role of the hippocampus during implicit learning, consistent with recent suggestions that this area is a rapid supramodal learner of arbitrary or higher-order associations in the sensory environment^3,16,28,32,33,40–42,46,80,81^. In a recent iEEG study presenting 12 syllables within an auditory stream, Henin et al.^16^ observed that TPs are encoded in lower-order areas of the superior temporal plane and not in the hippocampus, which uniquely represented the identity (i.e., the specific higher-order chunk such as a word) of their sequences. Therefore, the hippocampus did not appear to engage in forming the neural representation of TPs but performed operations that built upon them. On the contrary, we found the hippocampus to be the main contributor among the cortical areas in encoding TPs. These differences might emerge because our study used passive listening with pure tones, while Henin et al.^16^ used active listening with syllables. Our results fit well with previous studies indicating the hippocampus’ fundamental role in statistical learning and encoding stimuli uncertainty, both attended and unattended^3,16,28,32,33,40–42,44,46,79–82^. According to that, the hippocampus might operate differently depending on task demands. By its domain-general learning mechanisms, possible hippocampal involvement could comprise indirect modulation of lower-level sensory areas or direct computations of hippocampal representations^28,32^.

We also observed sensitivity to transitions between events in the inferior frontal cortex. Evidence of inferior frontal involvement in statistics-driven learning processes is sparse^28,33,42^ and mainly relies on explicit learning studies using fMRI^8,45^. However, it is commonly described in the deviance detection literature, where a role of a higher hierarchical node is attributed to this region^69,70,72^. Evidence from non-human primates iEEG studies manipulating the predictability of events also supports this involvement by showing a spatially dispersed contribution of regions that includes the prefrontal cortex in both passive auditory^43^ and active visual paradigms^6^.

Notably, channels in the superior temporal plane showed the highest encoded information and a high TP sensitivity (Fig. 3d), suggesting a key role of the supratemporal plane in both the deviance detection and the implicit learning of transitions between salient auditory events. This is consistent with previous reports about this region being active in conditional statistical learning^17,33,45,46,83^. Thus, the perceptual processing of individual stimuli in low hierarchical areas might be strongly affected by learning temporal patterns in streams of stimuli^22,23,28,84^. This is possibly due to a local process, top-down modulations, or both. However, previous studies have shown that top-down signaling interacts with bottom-up signaling at all hierarchy levels^5,6,69,70^.

A less expected observation was the significant TP sensitivity of individual channels in the occipital lobe, indicating a contribution to TP encoding of the auditory stimuli. It has been shown that during auditory oddball and statistical learning paradigms, attentional processing can activate visual processing regions, which are typically engaged in the perception of visual objects^16,85,86^. When queried, all of our participants reported that they could focus on the reading material and did not pay attention to the tones. Hence, this leaves open whether this auditory occipital activation might also be observable during passive listening tasks and whether this is specific to the sensitivity of our HFA recording. Current evidence is sparse, but two previous studies on deviance detection during passive listening showed similar occipital effects using fMRI and scalp EEG^66,86^.

Our results on deviance detection suggest a main involvement of the superior temporal plane and posterior insula (Fig. 2b). Previous studies on auditory deviance detection using iEEG, MEG/EEG source localization, and fMRI have shown similar responses to deviants over the supratemporal plane. However, detailed information for the insular cortex is sparse^1,35,63–71^. In line with recent reports about its contribution to auditory processing^64,87^, we found that the posterior part showed larger encoded information than the anterior part. We also noticed that the ACC, middle frontal, and pre-central sulcus moderately engaged in change detection. Although not often observed in auditory experiments, activation of these regions has been previously reported in the context of pre-attentive oddball paradigms with frequency (or duration) deviants using EEG^66,66,67,88^ or fMRI^71,86^. In our study, the ACC contributes to auditory change detection but did not reach a significant sensitivity to TP, which is generally consistent with previous reports^66,88^. It is presumably more involved in cognitive control or error detection, such as recognizing global patterns^63,65^. In our pre-attentive paradigm, we speculate that the ACC monitors the high-level structure of individual deviant occurrences rather than the automatic TP encoding. Further, areas lower in the hierarchy are more sensitive to deviant tones, and conversely, higher hierarchy locations exhibit lower encoded information values (Fig. 2c). Interestingly, our results indicate that the encoding of deviants was not strictly confined to specific areas but was distributed across multiple brain regions in a hierarchically organized manner. This suggests that lower hierarchical levels, which show a preferential representation of the stimuli, are more sensitive to the different deviant tones. Together, these results coincide with studies on the hierarchical visual pathway, which suggested that expectation suppression scales positively with image preference^89^.

Understanding the biophysical basis of deviant HFA responses to deviant stimuli is essential for interpreting neural dynamics. Responses to deviants are complex, involving shifts in timing, synchrony, or amplitude, with HFA capturing these nuances closely related to population-level spiking activity^48,64^. Recent studies using laminar multielectrode recordings in the sensory cortex of monkeys have identified two components of evoked HFA: an “early-deep” response, closely linked with multi-unit activity (MUA), and a “late-superficial” response, more apparent near the cortical surface with minimal association to MUA. The early-deep HFA corresponds with neuronal firing, while the late-superficial HFA likely represents dendritic activity independent of direct spiking^90^.

Predictive coding models posit that the brain predicts incoming stimuli and updates expectations based on deviations. Responses to deviants vary by temporal context, stimulus salience, and synaptic plasticity, affecting different neural network nodes, including the hippocampus^91^, which extends its role into auditory processing. Predictability has been shown to modulate the sharpening of representations in the sensory cortex. Reduced BOLD-fMRI responses have been observed for expected stimuli, while multi-voxel pattern analysis (MVPA) indicated higher classification accuracy and representational content^92^. While similar patterns could be anticipated in our HFA signals due to their correlation with fMRI, our observations show higher encoded information for deviants. This contrast may arise from our approach focusing on individual channels rather than using MVPA.

In the hippocampus, the interplay between excitability and inhibition influences neural firing, which is essential for implicit auditory statistical learning. Predictive coding models suggest that heightened excitability aligns with increased firing rates in response to TPs between stimuli. Conversely, inhibitory processes can temporally adjust neural firing patterns, allowing neurons to better respond to unexpected changes in stimuli. These adjustments help the brain manage unpredictability, ultimately contributing to the learning of new auditory patterns^93,94^.

Encoded information, as an information-theoretic measure, can capture non-linear effects, reflecting changes in neural complexity and predictability^62^ which indicate adaptive neural responses crucial for managing environmental variability—fundamental to predictive coding principles.

Our investigation of deviant HFA responses aligns with^95^, who demonstrated a hierarchy in predictive coding during speech processing. Their findings showed cortical areas predicting representations over multiple timescales, improving language model mapping to brain activity. This hierarchical approach complements our investigation by suggesting a broader framework where the hippocampus acts as a dynamic predictive map^96^, highlighting its crucial role not only in auditory processing but also in integrating complex predictions across temporal scales. These insights underscore the brain’s sophisticated ability to dynamically process and respond to unpredictable stimuli, enhancing our understanding of implicit auditory statistical learning mechanisms.

In our present study, we focused on the analysis of HFA, given that it captures swift fluctuations in iEEG. Aside from HFA, it might be especially worthwhile to consider lower frequency bands (e.g., alpha or beta) because these bands presumably carry information of predictions^5,6,97^. However, because iEEG represents the population activity of spiking neurons, it may miss less prominent activity patterns of a minority of neurons^1^. To make statements about dynamic TP sensitivity, it might be worth computing TP estimates given a limited integration window. This would allow us to determine whether specific regions are only active during a limited period. In the same vein, conducting a connectivity analysis would allow the investigation of the brain dynamics of this implicit statistical learning. While both of these points sound highly interesting, their implementation is non-trivial for the given high-dimensional sequence or iEEG recordings.

Our work provides a comprehensive picture of neural correlates of implicit statistical learning, which, before, were bundled together from multiple studies^28,33,46^. Additionally, our setup shares similarities with language learning studies. Yet, the implications of our findings may be limited because our paradigm is implicit and employs pure tones. One possibility to account for this is to replace pure tones with syllables or chunks of sounds. Also, given the presumably different roles of brain regions during implicit and active learning tasks^16,28^, active exposure to our sound train could potentially allow a more direct comparison between brain regions or language learning studies.

Having ascertained implicit learning analytically through algorithmic information theory and having determined neural substrates that imply a cortical network of brain regions, we are now in the position to explore its underlying mechanisms and regional influences further. Specifically, adding lower frequency bands to our analysis would enable us to disentangle the distinct roles in information encoding and predictability signaling of sensory inputs. While having a lower HFA, evoked responses to predictable events might exhibit a higher alpha or beta activity^5,6,43^. Accordingly, in the case of more frequently occurring and, thus, more predictable transitions, there might be an alternative cascade of regions anchored in higher cortical areas. In that respect, it might be worthwhile to evaluate the pre-onset sound interval of event responses, phase-amplitude coupling, or connectivity across ROIs.

Taken together, direct brain recordings reveal continuous encoding of structure in random unattended stimuli. While automatically assessing the deviance of events, the brain simultaneously identifies patterns by encoding conditional relations between events, supporting statistical learning and predictive coding frameworks. This implicit process involves, in addition to the hippocampus, inferior frontal cortices, pure sensory areas, and other cortical regions.

## Methods

### Stimuli

An unattended listening task following a multi-dimensional auditory oddball paradigm (Optimum-1) was used^69,70,98^. The implicit task consisted of a standard and five different deviant tones (Fig. 1). Standard events had a duration of 75 ms with 7 ms up and down ramps and consisted of three sinusoidal partials of 500, 1000, and 1500 Hz. Deviants varied relative to the standard in the perceived sound-source location (left or right), intensity (${\pm 6}$  dB), frequency (550, 1100, and 1650 Hz or 450, 900, and 1350 Hz), gap (25  ms silence in the middle), or by a shortened duration ($\nicefrac {1}{3}$ or 25 ms shorter). Thus, there were two stimuli versions for location, intensity, and frequency deviants. During the sequence, each standard tone was followed by a deviant. The deviant tone type was set up such that within five consecutive deviants, each of the five types was presented once. In successive sets, the same deviant type did not repeat from the end of one to the beginning of another. For the three deviants that had two stimulus versions, each version occurred equally often (P = 0.5). Except for deviants varying in duration, all tones had a duration of 75 ms and were presented every 500 ms in blocks of 5 m consisting of 300 standards and 300 deviants. At the beginning of each block, 15 standards were played. Participants were asked not to pay attention to the sounds while reading a book or magazine to capture automatic, stimulus-driven processes. They completed 3 to 10 blocks, providing at least 1800 trials. Tones were presented through headphones using Psychtoolbox-3^99^.

### Participants

We recorded data from 22 (self-reported) normal-hearing adults with drug-resistant epilepsy who were potential candidates for resective surgery of epileptogenic tissue (mean age 32 years, range 19 to 50 years, 7 female). Patients underwent invasive intracranial electrocorticography (ECoG) or stereoelectroencephalography (SEEG) recordings as part of their pre-surgical evaluation. Intracranial electrodes were temporarily implanted to localize the epileptogenic zone and eloquent cortex. The number and placement of electrodes were guided exclusively by clinical requirements. Data were collected at El Cruce Hospital (n = 15) and Oslo University Hospital (n = 7). See Table S1 for further demographic and clinical characteristics of the patient cohort.

### Data acquisition

Pre-implantation structural MRI and post-implantation CT scans were acquired for each participant. ECoG or SEEG data were recorded using an Elite (Blackrock NeuroMed LLC, USA), a NicoletOne (Nicolet, Natus Neurology Inc., USA), or an ATLAS (Neuralynx, USA) system with sampling frequencies of 2000, 512, and 16000 Hz, respectively.

### Electrode localization

Post-implantation CT images were co-registered to pre-implantation MRI images using SPM12^100^. MRI images were processed using the FreeSurfer standard pipeline^101^, and individual cortical parcellation images were obtained through the Destrieux atlas^102^. Electrode coordinates were obtained with the iElectrodes Toolbox^103,104^. Anatomical labels were automatically assigned to each contact based on the Destrieux atlas using the aforementioned toolboxes and confirmed by a neurologist/neurosurgeon. Coordinates were projected to the closest point on the pial surface (within 3 mm) and then co-registered to a normalized space using surface-based spherical coregistration^105^.

### Signal-preprocessing

Monopolar intracranial EEG recordings were visually inspected, and channels or epochs showing epileptiform activity or other abnormal signals were removed. Signals from electrodes in lesional or later resected tissue locations were also excluded. Bipolar channels were computed as the difference between signals recorded from pairs of neighboring electrodes in the same electrode array. We refer to these bipolar channels as “channels”. Data were low-pass filtered at 180 Hz, and line noise was removed using bandstop filters at 50, 100, and 150 Hz. Data were then segmented into 2000 ms epochs (750 ms before and 1250 ms after tone onset) and demeaned. We manually inspected and rejected epochs after bipolar re-referencing. To eliminate any residual artifact, we rejected trials with an amplitude larger than 5 SD from the mean for more than 25 consecutive ms, or with a power spectral density above 5 SD from the mean for more than 6 consecutive Hz. An average of 35% of the trials were rejected, resulting in an average of 1592 trials analyzed per patient (range 728 to 3723). Data were resampled to 1000 Hz. Pre-processing and statistical analysis were performed in Matlab using the Fieldtrip Toolbox^106^ and custom code. To obtain the HFA, pre-processed data were bandpass filtered into eight consecutive bands of 10 Hz bandwidth ranging from 75 to 145 Hz. The Hilbert transform was then applied to each filtered signal to obtain the complex-valued analytic time series, and the modulus of these signals was computed to retain the analytic amplitude time series. Trials were baseline corrected (− 100 to 0 ms) for each frequency band, and then the bands were averaged, producing a single time series per trial. We then selected a 0 to 400 ms post-stimulus window to effectively capture the core neural responses to auditory stimuli, encompassing key sensory and cognitive processing phases^42,46,64,73^. This window aligns with the typical timeframe for observing the Mismatch Negativity (MMN), which reflects the brain’s automatic detection of unexpected changes in auditory input^2,11,64,98^. Finally, for each channel, all trial time series were divided by the standard deviation pulled from all trials in the baseline period. For more information, see^64^, Chap. 2.

### Encoded information

We estimated the information content of HFA responses by employing the concept of Algorithmic Information Theory as described in^56,57^. This theory is anchored in Algorithmic Complexity or Kolmogorov Complexity (K-complexity). Given an object, the K-complexity constitutes its ultimate compressed version or minimum description length, i.e., its absolute information content^60^. If the minimum description length is short (long), an object is characterized as “simple” (“complex”). Because it is not possible to compute the theoretically ideal K-complexity, it is often heuristically estimated, obtaining an upper-bound approximation. Possible estimation approaches are conventional lossless data compression programs, e.g., gzip^60,61^.

Based on the K-complexity, various metrics were derived. One instance is the Normalized Information Distance or its estimation counterpart, the Normalized Compression Distance (NCD). The NCD allows a comparison of different pairs of objects with each other and suggests similarity based on their dominating features (or a mixture of sub-features)^60,61^. For a pair of strings (*x*, *y*), the ${\text{NCD}}(x,y)$ is defined as

$$\begin{aligned} {\text{NCD}}(x,y) = \frac{C(xy)-\min (C(x),C(y))}{\max (C(x),C(y))}, \end{aligned}$$

with *C*(*xy*) denoting the compressed size of the concatenation of *x* and *y*, and *C*(*x*) and *C*(*y*) their respective size after compression^60,61^. Further, the NCD is non-negative, that is, it is $0 \le {\text{NCD}}(x,y)\le 1+\epsilon$, where the $\epsilon$ accounts for the imperfection of the employed compression technique. Small NCD values suggest similar objects, and high values suggest rather different objects.

Labeling its implementation for neurophysiological data, *encoded information*, we utilized the complexity-based NCD measure to quantify information-content-based differences in neurophysiological recordings. More precisely, we defined single-trial *encoded information* values for each deviant response by computing the NCD measure between each HFA deviant response and the mean HFA standard response (for each channel; Fig. 1). Using a channel-specific mean response enables a common shared reference signal across deviant-to-standard comparisons.

In general, a compressed version of an HFA response was obtained by first simplifying it by grouping its values into 128 regular intervals (bins) while keeping the temporal sampling rate unchanged. The bins covered equal distances and in a range between the global extrema of all considered signals. A signal value $\text{X}(t)$ at time point *t* is closest to the bin $\text{Q}\in \mathbb {N}$ with $1 \le Q\le 128$. The value for the time point t used for the integer representation is $\text{Q}$. For example, consider the signal *x* consisting of nine random values

$$\begin{aligned} x&=\begin{pmatrix} 3.47&2.14&2.55&- 0.18&2.85&1.05&1.20&2.94&1.59 \end{pmatrix}^\intercal . \end{aligned}$$

Using 12 bins that cover equal distances and in a range between the global extrema, the following (ascending) edges result:

$$\begin{aligned} \text {edges}&= \left( \begin{array}{*{13}{c}} -\infty&\, 0.12&0.43&\, 0.73&\, 1.04&\, 1.34&\, 1.65&\, 1.95&\, 2.26&\, 2.56&\, 2.87&\, 3.17&\, \infty \end{array}\right) ^\intercal \end{aligned}$$

Note that on each end, $\infty$ is added to account for machine precision. Assigning each value of the signal to the closest edge then leads to the vector

$$\begin{aligned} x_{{\text{bin}}}&=\begin{pmatrix} 12&8&9&1&10&5&5&11&6 \end{pmatrix}^\intercal . \end{aligned}$$

This means that each element of x is mapped to a corresponding value in the binning vector “edges”. The resulting binned vector x$_\text {bin}$ is then compressed, yielding its compressed form C(x). The compression proceeded through a compression routine based on Python’s standard library with gzip. To account for the differences in auditory sound processing across channels, trial-specific encoded information values were normalized in terms of the channel mean of encoded information for the TP sensitivity analysis. For further details, see information and^56,57^, where we validated our approach.

### Transitional probability

We estimated the conditional statistics describing the inter-sound relationship with an ideal observer model: Specifically, we computed the TPs between adjacent deviant tones (disregarding standard tones). The TP of a deviant was determined by estimating their maximum-likelihood^14,25,26,107^

$$\begin{aligned} {\text{TP}}=\text{P}(\text{Y}|\text{X})=\frac{{\text{frequency}}({\text{XY}})}{{\text{frequency}}(\text{X})}, \end{aligned}$$

where the frequency(XY) of X$\rightarrow$Y transitions is normalized by the overall frequency of deviant X up to the current trial. TPs were estimated for each event-to-event combination X or Y. For each time point, the resulting TPs were then stored in a TP matrix (stochastic matrix of size $\mathbb {R}^{8\times 8}$).

### Anatomical hierarchy

Human T1w/T2w maps were obtained from the Human Connectome Project^76^. The maps were then converted from the surface-based CIFTI file format to the MNI-152 inflated cortical surface template with Workbench Command^108^. The structural neuroimaging maps are suggested to be a measure sensitive to regional variation in cortical gray-matter myelin content^74^. One function of myelin might be to act as an inhibitor of intracortical circuit plasticity. Early sensory areas may require less plasticity, hence more myelination, and hierarchically higher association areas, in turn, have less myelination, presumably enabling higher plasticity^109^. Accordingly, through an inverse relationship, T1w/T2w maps might serve as a non-invasive proxy of anatomical hierarchy across the human cortex. The anatomical hierarchy can be defined as a global ordering of cortical areas corresponding to characteristic laminar patterns of interareal projections^5,74,75^. To work directly with the hierarchy order, T1w/T2w maps were inverted and normalized to the value range of our data set.

### Statistical analysis

For the statistical analysis, the first 30 trials of each recording block were disregarded. By that, we aimed to exclude the initial phase of the experiment that potentially biases our correlation analysis. To estimate the TP sensitivity of a channel, all the eight distinct tone types were collapsed into one regressor, maximizing power. Subsequently, robust linear regression between the encoded information and the corresponding TP values was performed (Figs. 1 and 3a). Due to sparsity, TP values greater than 0.7 were excluded. We computed p-values by performing surrogate data testing for uncorrelated noise on the regression models. Null distributions of the regressor variables (encoded information and TPs) were generated by building shuffled surrogates (Fig. S2). Finally, the threshold for statistical significance was set to 0.05, and a false discovery rate (FDR) adjustment was applied to the p-values (FDR=0.05) to correct for multiple comparisons.

## Electronic supplementary material

Below is the link to the electronic supplementary material.

Supplementary Information.

## Data Availability

The cleaned and epoched HFA recordings, normalized electrode coordinates, materials for the experimental scripts and stimuli, and custom analysis code is available at https://osf.io/4t9c5. Due to the confidential nature of the data, the raw patient data analyzed for the current study are not publicly available. Our ethical approval conditions do not permit the public archiving of study data. Readers seeking access to the data should contact the author Alejandro Blenkmann, Department of Psychology, University of Oslo; the Research Ethics Committee of El Cruce Hospital, Argentina; and the Regional Committees for Medical and Health Research Ethics, Region North Norway. Requests must meet the following conditions to obtain the data: a collaboration agreement, a data-sharing agreement, and formal ethical approval.
