# Supplementary material for: Direct brain recordings reveal implicit encoding of structure in random auditory streams
Source: Sci Rep. 2025 Apr 27;15:14725. doi: 10.1038/s41598-025-98865-5 (PMC12034823; doi:10.1038/s41598-025-98865-5)
Supplement: Supplementary file 1 — Supplementary Information. [file 41598_2025_98865_MOESM1_ESM.pdf]

# Direct brain recordings reveal implicit encoding of structure in random auditory streams

## Supplementary Information

Julian Fuhrer, Kyrre Glette, Jugoslav Ivanovic, Pål Gunnar Larsson, Tristan Bekinschtein, Silvia Kochen, Robert T. Knight, Jim Tørresen, Anne-Kristin Solbakk, Tor Endestad and Alejandro Blenkmann

In this document, first, a detailed description of the regions of interests (ROIs) is supplied. Second, the significance testing by use of surrogates is demonstrated (Fig. S2). Then, statistically significant differences of the encoded information measure across ROIs is presented (Fig. S1), followed by the significance ratio (number of significant to total channels) across ROIs and subjects (Fig. S3). In Fig. S4, the distribution of the Pearson correlation coefficients is demonstrated. Subsequently, the deviant-specific encoded information across ROIs is depicted (Fig. S5), and, lastly, the effect of the normalization step is visualized (Fig. S6).

## Contents

1 Subject Summary 2
2 Regions of Interest 3
3 Mean Encoded Information Across ROIs 4
4 Significance Testing 5
5 Significance Ratio Across ROIs 6
6 Pearson Correlation Coefficient 7
7 Deviant-specific Encoded Information Across ROIs 8
8 Normalization of Encoded Information for TP sensitivity 9
9 Encoded Information 10
10 Comparing channel-wise Variance in Standard HFA response to Encoded Information 13
11 TP sensitivity across ROIs using Mixed-Effects Models 14
References 15

# 1 Subject Summary

| Subject | Age | Sex | Handed-ness | Epilepsy Start (y) | MRI Lesion | Number of Impl. Electrodes (not all recording) | Recording Type | Hemisphere | Comments | Epileptogenic Zone |
|---|---|---|---|---|---|---|---|---|---|---|
| Subject01 | 20 | M | R | 10 | FCD in the left occipitotemporal region, temporal pole alteration, and EHI. | 23 | SEEG | Left | NA | Left mesial temporal |
| Subject02 | 22 | F | R | 17 | no | 44 | SEEG | Right | Hypometabolism in Right insula (PET) | Right insula |
| Subject03 | 19 | M | R | 14 | no | 47 | SEEG | Bilateral | Hypometabolism in Left temporal (PET) | Left hippocampus |
| Subject04 | 49 | F | R | 17 | Right precentral region, occipital cuneus, supracalcarine and infracalcarine areas, inferior parietal lobe, and precuneus. | 56 | SEEG | Bilateral | Hypometabolism in Right Temporo-Parietal-Occipital (PET) | Right cuneus, occipital, supracalcarine, and posterior hippocampus |
| Subject05 | 19 | M | R | 18 | no | 49 | SEEG | Bilateral | NA | Left and right hippocampus |
| Subject06 | 25 | M | L | 15 | Right subependymal heterotopia parietal inferior - occipital medial | 45 | SEEG | Right | NA | Right cuneus - pericalccarine |
| Subject07 | 37 | M | R | 24 | Left superior - middle frontal white matter alteration | 47 | SEEG | Bilateral | NA | Left mesial temporal |
| Subject08 | 33 | M | R | 7 | FCD frontal lateral left | 42 | SEEG | Left | NA | Left middle frontal gyrus and pars opercularis |
| Subject09 | 23 | F | R | 6 | Bilateral frontal lobe lesions with mild atrophy | 68 | SEEG | Right | NA | Right superior parietal |
| Subject10 | 38 | M | L | 5 | Left Frontoparietal operculum and insula | 44 | SEEG | Bilateral | NA | Frontal operculum and insula |
| Subject11 | 33 | F | R | 1 | no | 68 | SEEG | Bilateral | NA | Right middle temporal |
| Subject12 | 20 | M | R | 6 | Right parieto-occipital FCD | 120 | ECoG+SEEG | Right | NA | Right occipital and parietal |
| Subject13 | 44 | F | R | NA | no | 62 | ECoG+SEEG | Left | NA | Left mesial temporal |
| Subject14 | 29 | M | R | 3 | Bilateral hippocampal sclerosis | 38 | SEEG | Bilateral | NA | Left mesial temporal |
| Subject15 | 24 | F | R | 5 | no | 64 | ECoG+SEEG | Left | Right hemiparesis | Left superior frontal gyrus |
| Subject16 | 27 | M | R | 24 | Left Wernicke | 101 | SEEG | Bilateral | NA | Next to left primary sensory cortex, extending to trigone |
| Subject17 | 34 | F | R | 11 | Left parietal | 165 | SEEG | Bilateral | NA | Left parietal |
| Subject18 | 35 | M | R | 26 | Right temporal | 110 | SEEG | Right | NA | Right temporal |
| Subject19 | 48 | M | R | 14 | Right temporal | 172 | SEEG | Right | NA | Suspected on right parietal |
| Subject20 | 25 | M | R | 10 | Left frontal glioma | 170 | SEEG | Left | NA | Left frontal |
| Subject21 | 52 | M | R | 8 | Small frontal dysplasia (MFG) | 164 | SEEG | Right | NA | Right Middle Frontal Sulcus |
| Subject22 | 50 | F | R | 27 | Right hippocampal resection | 147 | SEEG | Bilateral | NA | Left Hippocampus |

## 2 Regions of Interest

**Table S2:** Anatomical parcellations of the ROIs after the Destrieux atlas.

| ROI | Short name | Long name |
|---|---|---|
| Superior temporal plane | G_temp_sup-Lateral | Lateral aspect of the superior temporal gyrus |
| | G_temp_sup-G_T_transv | Anterior transverse temporal gyrus (of Heschl) |
| | G_temp_sup-Plan_tempo | Planum temporale or temporal plane of the superior temporal gyrus |
| | S_temporal_transverse | Transverse temporal sulcus |
| Lateral temporal cortices | G_temp_sup-Lateral | Lateral aspect of the superior temporal gyrus |
| | G_temporal_inf | Inferior temporal gyrus |
| | S_temporal_sup | Superior temporal sulcus (parallel sulcus) |
| | S_temporal_inf | Inferior temporal sulcus |
| | G_temporal_middle | Middle temporal gyrus |
| Superior frontal cortices | S_front_sup | Superior frontal sulcus |
| | G_front_sup | Superior frontal gyrus |
| Middle frontal cortices | S_front_middle | Middle frontal sulcus |
| | G_front_middle | Middle frontal gyrus |
| Inferior frontal cortices | S_front_inf | Inferior frontal sulcus |
| | G_front_inf-Opercular | Opercular part of the inferior frontal gyrus |
| | G_front_inf-Orbital | Orbital part of the inferior frontal gyrus |
| | G_front_inf-Triangul | Triangular part of the inferior frontal gyrus |
| | Lat_Fis-ant-Horizont | Horizontal ramus of the anterior segment of the lateral sulcus (or fissure) |
| | Lat_Fis-ant-Vertical | Vertical ramus of the anterior segment of the lateral sulcus (or fissure) |
| Pre-central sulci | G_precentral | Precentral gyrus |
| | S_central | Central sulcus (Rolando's fissure) |
| | S_precentral-sup-part | Superior part of the precentral sulcus |
| | S_precentral-inf-part | Inferior part of the precentral sulcus |
| | G_and_S_paracentral | Paracentral lobule and sulcus |
| | G_and_S_subcentral | Subcentral gyrus (central operculum) and sulci |
| Anterior cingulate cortices | G_and_S_cingul-Ant | Anterior part of the cingulate gyrus and sulcus (ACC) |
| | G_and_S_cingul-Mid-Ant | Middle-anterior part of the cingulate gyrus and sulcus (aMCC) |
| Anterior insula | S_circular_insula_ant | Anterior segment of the circular sulcus of the insula |
| | S_circular_insula_sup | Superior segment of the circular sulcus of the insula |
| | G_insular_short | Short insular gyri |
| Posterior insula | S_circular_insula_inf | Inferior segment of the circular sulcus of the insula |
| | G_Ins_lg_and_S_cent_ins | Long insular gyrus and central sulcus of the insula |

# 3 Mean Encoded Information Across ROIs

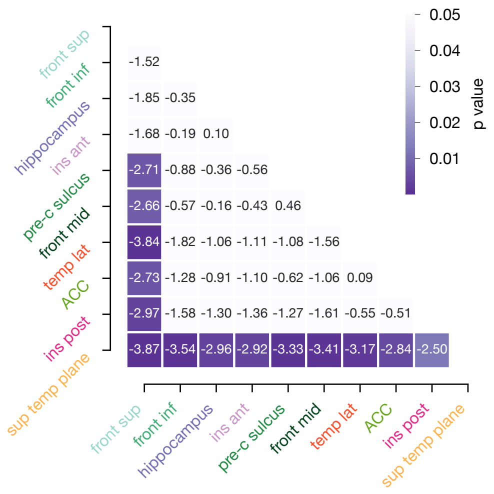


**Figure S1:** Matrix of z-values representing individual statistical differences of the encoded information measure (two-tailed pairwise Mann–Whitney–Wilcoxon tests) across ROIs.

# 4 Significance Testing

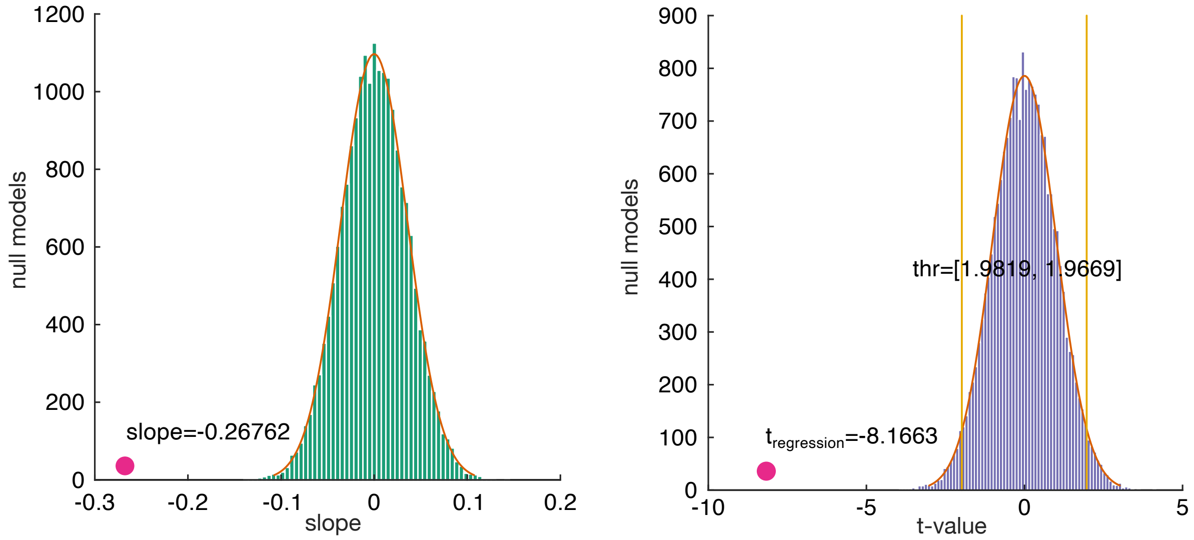


**Figure S2:** Significance testing through surrogates for an example channel. By randomly shifting the elements of the regression array that consists of encoded information and transition probalbities (TPs), 2e4 null models are generated. The hypothesis that the regression correlates noise can be rejected, when the respective t value is above or below the 2.5 % or 97.5 % quantile of the null model distribution.

# 5 Significance Ratio Across ROIs

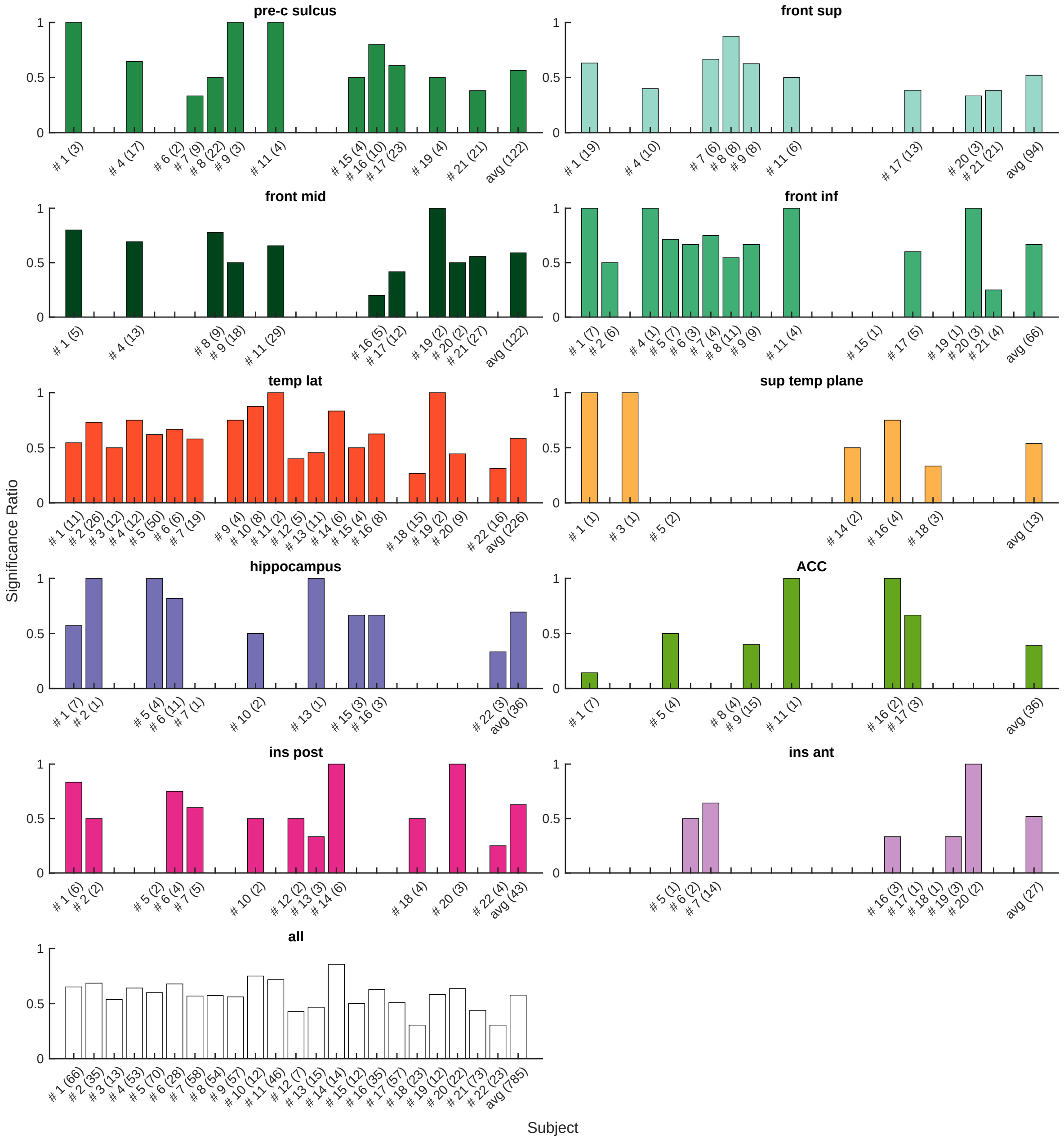


**Figure S3:** Distribution of the significant channels across subjects. 485 out of 785 channels show a significant slope after adjusting for channels-wise multiple correction and surrogate testing. On the x-label, the number in brackets shows the amount of channels each subject has in the respective ROI. The ratio itself is defined as the number of significant channels to total number of channels. On the bottom left, all ROIs are taken together.

# 6 Pearson Correlation Coefficient

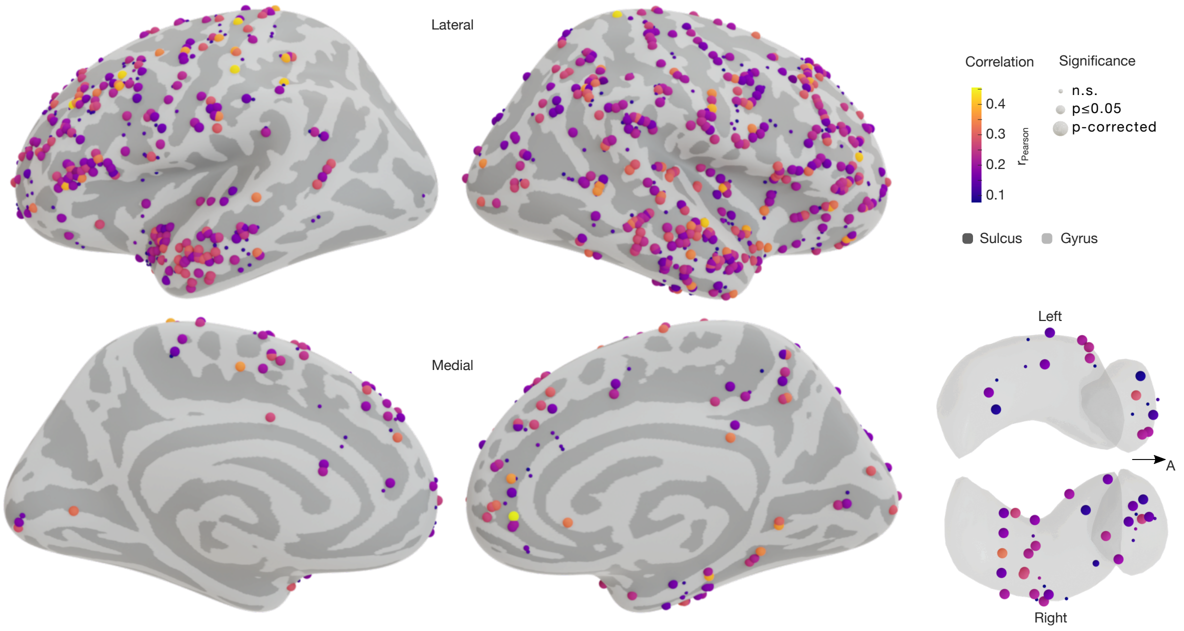


**Figure S4:** Inflated brain model with lateral and medial views of the right and left hemispheres and a superior view of the amygdala and hippocampus. Each sphere represents a channel projected onto the surface with the colors indicating its Pearson correlation coefficient resutling from the regressio of encoded information to TPs. The size of the spheres indicates the p-value corresponding to the performed regression. The p-values are divided such that each interval contains 1/4 of the p-value set.

## 7 Deviant-specific Encoded Information Across ROIs

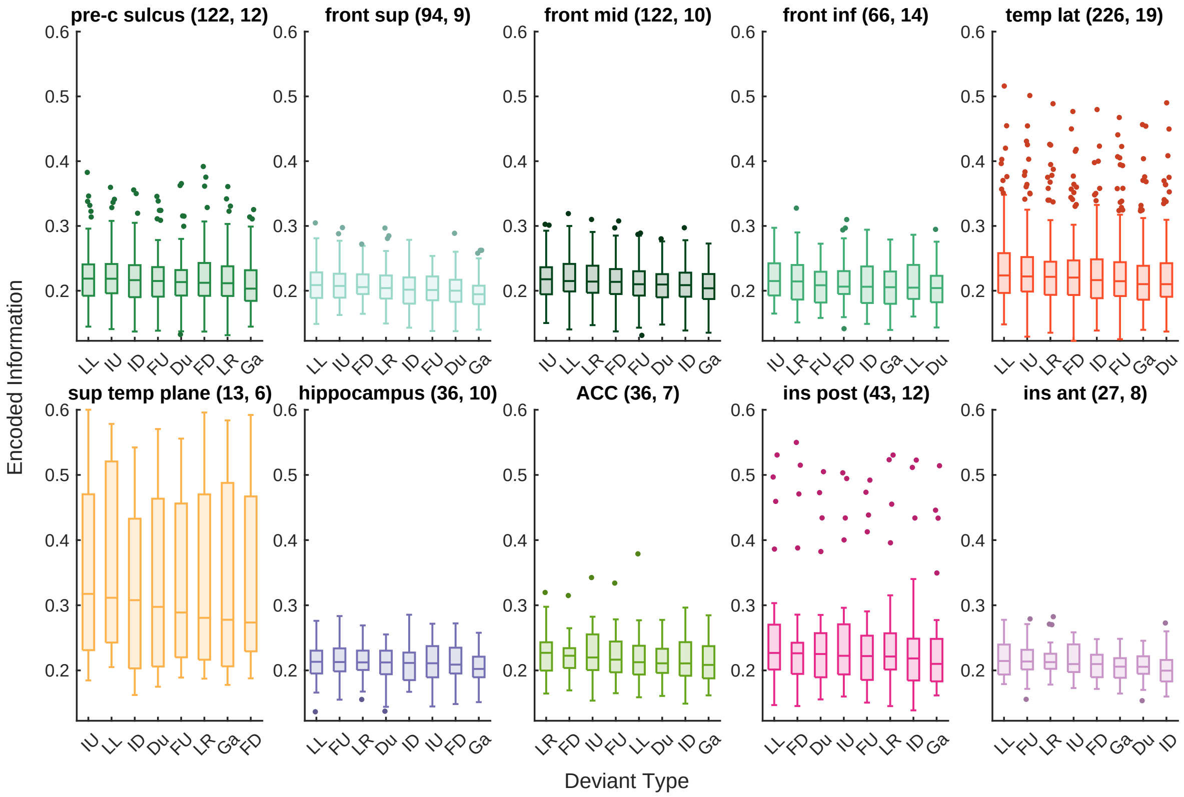


**Figure S5:** Distribution of the encoded information measure across ROIs for individual deviant types. In the axes labels ”LL/R” stands for location left/right, ”IU/D” for intensity up/down, ”FD/U” for frequency down/up, ”Du” for duration and ”Ga” for gap. In the titles, the term after each ROI name indicates the number of channels (first) and subjects (second). Statistical analysis showed no significant differences in the *encoded information* of specific deviant types in any of the areas but for superior frontal area with differences between the deviant types of ”location left”, ”intensity up”, and ”frequency down” to ”gap” (two-tailed pairwise Mann–Whitney–Wilcoxon tests, FDR corrected, $p \leq 5.30e{-4}$, $z \geq 3.5$).

## 8 Normalization of Encoded Information for TP sensitivity

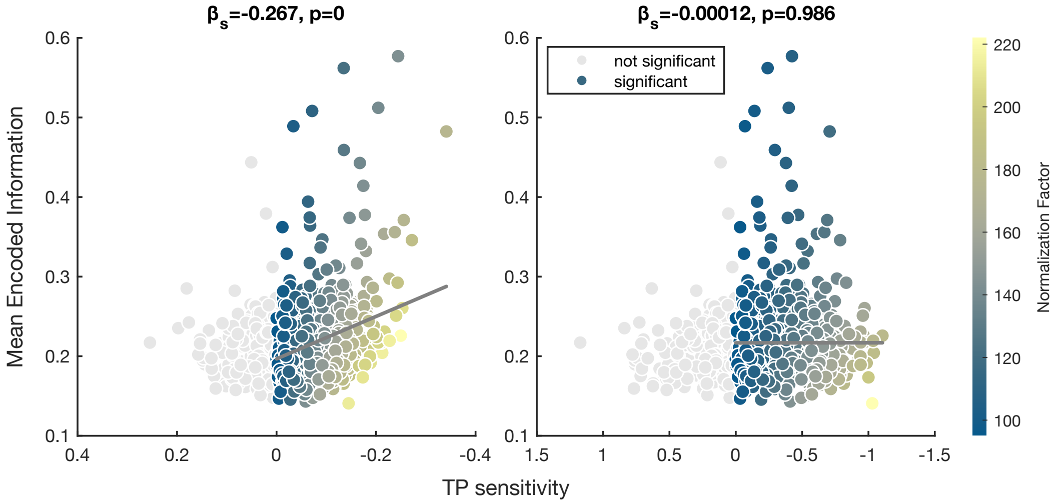


**Figure S6:** Relation between the mean encoded information and TP sensitivity before (left) and after (right) normalization. Without the channel-wise normalization of the encoded information measure, there is a significant positive correlation between the mean encoded information and TP sensitivity (left graph; linear mixed-effects model with random effects for subjects: $y = \beta_0 + \beta_1 x + b_0 + \epsilon$, with the mean encoded information $y$, the TP sensitivity $x$, the random effect for subjects $b_0 \sim N(0, \sigma_b^2)$ and the observation error $\epsilon \sim N(0, \sigma^2)$; $\beta_0 = 0.19$, 95% CI [0.19, 0.20], $\beta_1 = 0.28$, 95% CI [0.22 0.33], $p_{\beta_1} = 0$, $\sigma_b = 1.39\text{e}{-2}$, 95% CI [9.46e−3, 2.04e−2], $\epsilon = 4.09\text{e}{-2}$, 95% CI [3.89e−2, 4.29e−2]). As we are interested in the effect that solely can be traced back to the variance of the TPs, we adjusted for that by normalizing each encoded information value for each trial by its respective channel mean. Applying this step corrects for any correlation between mean encoded information and TP sensitivity (right graph). Consequently, by regressing encoded information with TPs, a signifiant slope only emerges if there is a real effect between the two regressors.

# 9 Encoded Information

## Using Information Theory to Decode Neural Dynamics

At its core, neural systems constantly encode, integrate, and store information originating from the steady stream of input to the sensory organs. Information theory has been widely applied to neuroscience research as it offers multivariate analysis tools, is not bound to a single type of data, is model-independent (i.e., does not require assumptions about the data itself), and can capture nonlinear interactions (1–3).

## Application of Algorithmic Complexity

Specifically, principles of Algorithmic Information Theory, or more precisely, Algorithmic Complexity or Kolmogorov Complexity (K-complexity) have been employed to discriminate between states of consciousness measured with EEG, iEEG, MEG, or fMRI recordings (4–7). It was independently developed by R.J. Solomonoff A.N. Kolmogorov and G. Chaitin (8–10) and enables the estimation of information content or complexity of an individual object by measuring the length of its shortest description (11). For example, the sequence

ABABABABABABABABABABABABAB

can be described as “AB repeats 27 times”. The structure of the sequence

AABBABBBBAABBAAABAAAABBBAA

on the other hand, is less clear. Intuitively, there appears to be no simple description such that the second sequence is more complex or exhibits a higher information content than the first sequence. More formally, K-complexity is the ultimate compressed version or minimum description length of an object or its absolute information content(11). If the minimum description length is short (long), an object is characterized as “simple” (“complex”).

## Estimating Komogorov Complexity

As evident from the two example sequences, there can be scenarios where it is rather difficult to infer a rule that describes an object with minimum description length. In general, it is not possible to compute the theoretically ideal K-complexity. Therefore, when applying this theory, an estimate needs to be chosen, yielding an upper-bound approximation (an estimate of the description length is always longer than the ultimate theoretically possible minimum description length). Possible estimation approaches are conventional lossless data compression programs such as gzip, bzip2, or the Lempel–Ziv–Markov chain algorithm (LZMA) (11, 12).

## Neuroscientific Use-Case and Methodology

In neuroscientific studies, it is common to have two or more task conditions (for example, control and test conditions) that are compared with each other. The K-measure so far yields an estimate of the absolute information content of a recording. Fortunately, based on the K-complexity, various metrics were derived that allow a pairwise comparison between two objects. One instance is NCD allowing for the comparison of different pairs of objects with each other and suggests similarity based on their dominating features (or a mixture of sub-features) (11, 12). This measure has been applied to, for example, cluster analysis, virology (even to analyze the SARS-CoV-2 virus by two of the original authors behind this measure (13), language, music, and literature (11). For a pair of strings (x,y), NCD is defined as

$$\mathrm{NCD}(x,y) = \frac{C(xy) - \min(C(x), C(y))}{\max(C(x), C(y))}, \tag{1}$$

with $C(xy)$ denoting the compressed size of the concatenation of $x$ and $y$, and $C(x)$ and $C(y)$ their respective size after compression (11, 12, 14–16). Further, the NCD is non-negative, that is, it is $0 \leq$

$\mathrm{NCD}(x, y) \leq 1 + \epsilon$, where the $\epsilon$ accounts for the imperfection of the employed compression technique. Smaller NCD values suggest similar objects. Higher values suggest rather different objects. There is an intuitive interpretation of NCD (15, 16). For instance, given $C(y) \geq C(x)$ the compression distance becomes

$$\mathrm{NCD}(x, y) = \frac{C(xy) - C(x)}{C(y)} . \tag{2}$$

Accordingly, it becomes the ratio between the improvement of compressing y using x as a previously compressed "database" relative to compressing y from scratch (11, 15). Using the definition of the conditional compressed information $C(y|x) = C(xy) - C(x)$, it can be also written as

$$\mathrm{NCD}(x, y) = \frac{C(y|x)}{C(y)} , \tag{3}$$

that is, it is the ratio of the information $x$ about $y$ to the information in $y$ (15).

## Implementation for Neurophysiological Data

Labeling its implementation for neurophysiological data, *encoded information*, we utilized the complexity-based NCD measure to quantify information-content-based differences in neurophysiological recordings. In our study, the input strings were brain signals after some data transformation steps:

The general procedure to obtain a compression version C(x) of a signal x was to first represent the continuous signal by grouping its values into discrete steps (bins). Accordingly, the signal was simplified by reducing its resolution along the y-axis for example into 127 bins. The bins covered equal distances and were in a range between the global extrema of all recorded signals (e.g., the global extrema of a channel). After simplifying the signal, a compressor then received the indices of the bins that contained the elements of the signals (4, 5). That is, each sample is mapped to a value of the binning vector. This index vector then underwent compression through a routine based on Python's standard library and gzip. At this point, it is worth pointing out that the length of the index vector is invariant to the number of bins. This is because the length of the signal is not changed during the binning procedure, but only the resolution along the y-axis. Consequently, each value within the index vector represents a signal sample in ascending order. Thus, the input length of the compressors remains constant.

For example, consider the signal x consisting of nine random consecutive samples

$$x = \begin{pmatrix} 3.47 & 2.14 & 2.55 & -0.18 & 2.85 & 1.05 & 1.20 & 2.94 & 1.59 \end{pmatrix}^{\intercal} .$$

Using 12 bins that cover equal distances and in a range between the global extrema, the following (ascending) edges result:

$$\text{edges} = \begin{pmatrix} -\infty & 0.12 & 0.43 & 0.73 & 1.04 & 1.34 & 1.65 & 1.95 & 2.26 & 2.56 & 2.87 & 3.17 & \infty \end{pmatrix}^{\intercal}$$

Note that on each end, $\infty$ is added to account for machine precision. Assigning each value of the signal to the closest edge then leads to the vector

$$x_{\text{bin}} = \begin{pmatrix} 12 & 8 & 9 & 1 & 10 & 5 & 5 & 11 & 6 \end{pmatrix}^{\intercal} .$$

This means that each element of x is mapped to a corresponding value in the binning vector "edges". The resulting binned vector xbin is then compressed, yielding its compressed form C(x). Across the data sets considered, the median encoded information was 0.7414 ± 0.0528, the minimum is 0.5344 ± 0.1206, and the maximum was 0.9720 ± 0.0708. In the present study, we employed this approach by correlating the encoded information (i.e., NCD(HFA(deviant trial), HFA(mean(standard trial))) with the respective TP value through robust linear regression. We further performed a surrogate analysis to evaluate the robustness of our results by randomly shuffling the encoded information values per channel and performing robust regression for each shuffle. The resulting p value was then corrected for multiple comparisons across channels using FDR.

## Methods Comparison

Standard amplitude-based or power-based comparisons, such as t-tests and cluster-permutation tests, are valuable for establishing whether responses to deviant stimuli reliably differ from those to standard stimuli. However, these methods typically assume linear relationships and may not fully capture differences in nonlinear, higher-order temporal structures (1–3). In tasks where deviants vary subtly in their time-domain waveforms or exhibit pattern changes beyond simple amplitude shifts, traditional approaches may overlook important features.

Our encoded information (EI) approach, evaluated in detail in our previous studies (17, 18), addresses these limitations. We applied this measure to three different neurophysiological and synthetic data sets and compared it with conventional methods such as t-tests, mutual information (MI), advanced MI estimation through Gaussian Copula (GCMI; (3)), and neural frequency tagging (NFT). This comparison highlighted the ability of our method to uncover nuances that might be missed by approaches relying on linear models (18). Especially for HFA, the encoded information measure performs among the best across the different methods considered.

Similarly, cluster-based permutation tests, typically conducted at the level of regions of interest (ROIs), are based on linear assumptions related to power or amplitude at each time sample, which can lead to potential oversights of nonlinearities. Our method, though it effectively captures overall temporal dynamics, has a limitation in that it loses the fine-grained temporal dynamics within individual trials.

Consequently, while traditional methods are excellent for linear analyses, our encoded information metric provides a complementary tool, capturing complexities and nuances in the data that are crucial for understanding the full scope of brain responses during statistical learning tasks.

## Summary

Taken together, in our study, we correlated the encoded information (NCD between deviant and standard trials) with the respective TP value through robust linear regression. This analysis uncovered how variably the brain processes unexpected stimuli based on probabilistic expectations in the auditory sequence, represented by TPs. We further conducted surrogate analyses by shuffling encoded information values per channel and re-evaluating through regression. The resulting p-values were then used after adjusting for FDR across channels for follow-up analyses.

## 10 Comparing Variance in Standard HFA response to Encoded Information

Using the lme4 package in R version 4.4.3, we employed a linear mixed-effects model that regresses the channel-specific variance of HFA responses to standard tones on the channel-specific EI measure, with random effects for subjects. Outliers in both predictor and outcome variables were identified and removed using the interquartile range method to improve the robustness of the findings .
The results of this analysis revealed that as the variance in Standard HFA responses increases, the expected EI also rises (Estimate = 0.06650, Std. Error = 0.03055, t value = 2.177). Random effects showed minimal variance at the subject level (Variance = 0.0001340, Std. Dev. = 0.01158), indicating consistent responses across subjects. This finding supports the notion that variability in neuronal responses correlates with greater information encoding, highlighting the relevance of accounting for such variance in our analyses. Fig. S7 depicts the relationship between channel-wise variance in Standard HFA and channel-wise EI. This figure illustrates the positive association between these factors, confirming the importance of considering variance in Standard HFA responses in relation to EI. We believe this has enriched our understanding of how neural response variability can impact the encoding of information.

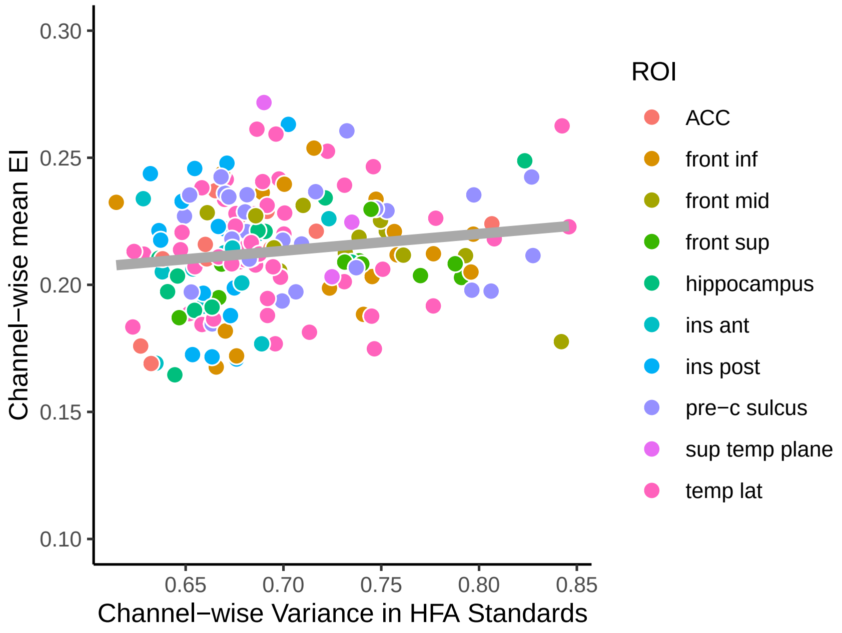


**Figure S7:** Relationship between channel-wise variance in standard HFA responses and channel-wise mean EI. Each point represents a channel, color-coded by ROI. The solid grey line indicates the predicted mean EI based on variance in standard HFA responses, highlighting a positive correlation. The data suggest that increased variability in standard HFA corresponds to greater information encoding, supporting the notion that dynamic neural responses correlate with enhanced information processing.

We would further like to point out that we normalized the EI single-trial values for each channel with the channel-specific mean EI value. As described in the manuscript and in Fig. S6, this normalization step aims to account for effects other than TP encoding, which might be influenced by considering the variance of standard responses. This step corrects for the effect of mean EI on TP sensitivity. Consequently, regressing EI with TPs produces a significant slope only if there is a real effect between the two variables.

# 11 TP sensitivity across ROIs using Mixed-Effects Models

Using the lme4 package in R version 4.4.3, we regressed TPs on EI across all ROIs and subjects with an interaction term for ROIs and a random effect for subjects. We further chose the ROI of “ACC” as a reference, showing the weakest TP sensitivity (Fig. 3d in the manuscript). The results of the mixed-effects model fit closely with our approach as can be seen from Fig. S8 and the model summary below. Notably, the hippocampus shows the greatest TP sensitivity (-0.21). Regarding the random effects, the variance associated with the subject-level intercept is small (2.297e-32), suggesting limited variability across subjects. We would like to mention that during pre-processing, all trials were baseline-corrected, which may account for inter-subject variation. Overall, this affirms the robustness of our findings, and we believe the current methodology remains valid and sufficient for our analysis.

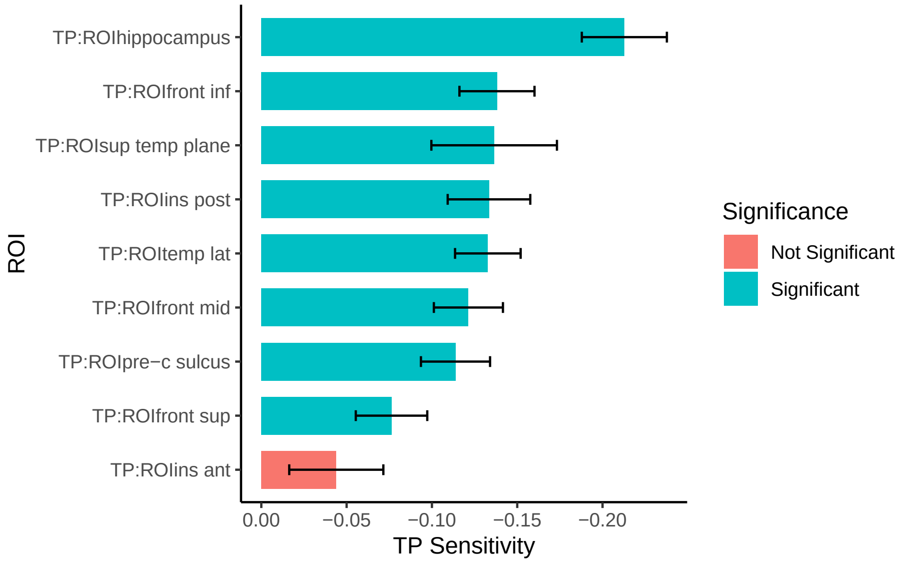


**Figure S8:** TP sensitivity across ROIs using a linear mixed-effects model with interaction terms for TP and ROIs, along with random effects for subjects. The analysis is based on the channel-specific EI while using ”ACC” as the reference ROI. Significant differences in TP sensitivity indicate how standard HFA measures vary across different ROIs.

**Table S3:** Mixed-Effects Model Results for TP Sensitivity Across ROIs

| **Effects** | **Estimate** | **Std. Error** | **t value** |
|---|---|---|---|
| | **Fixed Effects** | | |
| (Intercept) | 1.038112 | 0.004079 | 254.516 |
| TP | -0.180230 | 0.017862 | -10.090 |
| ROIhippocampus | 0.044988 | 0.005696 | 7.898 |
| ROIfront inf | 0.029334 | 0.005028 | 5.834 |
| ROIfront mid | 0.025674 | 0.004622 | 5.555 |
| ROIfront sup | 0.016074 | 0.004784 | 3.360 |
| ROIins ant | 0.009519 | 0.006316 | 1.507 |
| ROIins post | 0.028168 | 0.005528 | 5.096 |
| ROIpre-c sulcus | 0.024021 | 0.004625 | 5.194 |
| ROIsup temp plane | 0.028728 | 0.008393 | 3.423 |
| ROItemp lat | 0.028271 | 0.004389 | 6.441 |
| TP:ROIhippocampus | -0.212772 | 0.024931 | -8.534 |
| TP:ROIfront inf | -0.138120 | 0.022003 | -6.277 |
| TP:ROIfront mid | -0.121375 | 0.020239 | -5.997 |
| TP:ROIfront sup | -0.076315 | 0.020955 | -3.642 |
| TP:ROIins ant | -0.043948 | 0.027577 | -1.594 |
| TP:ROIins post | -0.133443 | 0.024207 | -5.513 |
| TP:ROIpre-c sulcus | -0.113813 | 0.020255 | -5.619 |
| TP:ROIsup temp plane | -0.136469 | 0.036797 | -3.709 |
| TP:ROItemp lat | -0.132771 | 0.019212 | -6.911 |
| | **Random Effects** | | |
| Subject (Intercept) | 2.297e-32 | - | 1.516e-16 |
| Residual | 4.996e-02 | - | 2.235e-01 |

# References


1. N. M. Timme and C. Lapish, “A tutorial for information theory in neuroscience,” *eNeuro*, vol. 5, no. 3, 2018.
2. E. Piasini and S. Panzeri, “Information theory in neuroscience,” *Entropy*, vol. 21, no. 1, 2019. [Online]. Available: https://www.mdpi.com/1099-4300/21/1/62
3. R. A. Ince, B. L. Giordano, C. Kayser, G. A. Rousselet, J. Gross, and P. G. Schyns, “A statistical framework for neuroimaging data analysis based on mutual information estimated via a gaussian copula,” *Human Brain Mapping*, vol. 38, no. 3, pp. 1541–1573, 2017.
4. J. D. Sitt, J.-R. King, I. El Karoui, B. Rohaut, F. Faugeras, A. Gramfort, L. Cohen, M. Sigman, S. Dehaene, and L. Naccache, “Large scale screening of neural signatures of consciousness in patients in a vegetative or minimally conscious state,” *Brain*, vol. 137, no. 8, pp. 2258–2270, 06 2014.
5. A. Canales-Johnson, A. J. Billig, F. Olivares, A. Gonzalez, M. d. C. Garcia, W. Silva, E. Vaucheret, C. Ciraolo, E. Mikulan, A. Ibanez, D. Huepe, V. Noreika, S. Chennu, and T. A. Bekinschtein, “Dissociable Neural Information Dynamics of Perceptual Integration and Differentiation during Bistable Perception,” *Cerebral Cortex*, vol. 30, no. 8, pp. 4563–4580, 03 2020.
6. M. Schartner, A. Seth, Q. Noirhomme, M. Boly, M.-A. Bruno, S. Laureys, and A. Barrett, “Complexity of multi-dimensional spontaneous eeg decreases during propofol induced general anaesthesia,” *PLOS ONE*, vol. 10, no. 8, pp. 1–21, 08 2015.
7. M. Schartner, A. Pigorini, S. A. Gibbs, G. Arnulfo, S. Sarasso, L. Barnett, L. Nobili, M. Massimini, A. K. Seth, and A. B. Barrett, “Global and local complexity of intracranial EEG decreases during NREM sleep,” *Neuroscience of Consciousness*, vol. 2017, no. 1, 01 2017.
8. R. Solomonoff, “A formal theory of inductive inference. part i,” *Information and Control*, vol. 7, no. 1, pp. 1–22, 1964.
9. G. J. Chaitin, “On the length of programs for computing finite binary sequences: Statistical considerations,” *J. ACM*, vol. 16, no. 1, p. 145–159, 01 1969.
10. A. N. Kolmogorov, “Three approaches to the quantitative definition of information,” *International Journal of Computer Mathematics*, vol. 2, no. 1-4, pp. 157–168, 1968.
11. M. Li and P. Vitányi, *An Introduction to Kolmogorov Complexity and Its Applications*, 3rd ed., ser. Texts in Computer Science. Springer New York, 2008.
12. M. Li, X. Chen, X. Li, B. Ma, and P. Vitanyi, “The similarity metric,” *IEEE Transactions on Information Theory*, vol. 50, no. 12, pp. 3250–3264, 2004.
13. R. L. Cilibrasi and P. M. B. Vitányi, “Fast phylogeny of sars-cov-2 by compression,” *Entropy*, vol. 24, no. 4, 2022.
14. R. L. Cilibrasi and P. M. B. Vitányi, “Clustering by compression,” *IEEE Transactions on Information Theory*, vol. 51, no. 4, pp. 1523–1545, 2005.
15. R. L. Cilibrasi, “Statistical inference through data compression,” Ph.D. dissertation, Institute for Logic, Language and Computation, Universiteit van Amsterdam, Plantage Muidergracht 24, 1018 TV, Amsterdam, Holland, 02 2007.
16. P. M. B. Vitányi, F. J. Balbach, R. L. Cilibrasi, and M. Li, *Normalized Information Distance*. Boston, MA: Springer, 2009, pp. 45–82.
17. J. Fuhrer, A. Blenkmann, T. Endestad, A.-K. Solbakk, and K. Glette, “Complexity-based encoded information quantification in neurophysiological recordings,” in *2022 44th Annual International Conference of the IEEE Engineering in Medicine & Biology Society (EMBC)*, 2022, pp. 2319–2323.
18. J. Fuhrer, K. Glette, A. Llorens, T. Endestad, A.-K. Solbakk, and A. O. Blenkmann, “Quantifying evoked responses through information-theoretical measures,” *Frontiers in Neuroinformatics*, vol. 17, 2023.
